# *Cryptococcus neoformans* and Other Opportunistic *Cryptococcus* Species in Pigeon Dropping in Saudi Arabia: Identification and Characterization by DNA Sequencing

**DOI:** 10.3389/fmicb.2021.726203

**Published:** 2021-10-11

**Authors:** Bashir Sirag, El-Shiekh Khidir, Mohammed Dumyati, Basam Sindi, Mahir Alsinnari, Hani Faidah, Abdalla Ahmed

**Affiliations:** ^1^Department of Microbiology, College of Medicine, Umm Al-Qura University, Makkah, Saudi Arabia; ^2^Laboratory Medicine Department, Faculty of Applied Medical Sciences, Umm Al-Qura University, Makkah, Saudi Arabia; ^3^Department of Medicine, National Guard Health Affairs, King Abdulaziz Medical City, Jeddah, Saudi Arabia; ^4^Department of Medicine, King Fahad Armed Forces Hospital, Jeddah, Saudi Arabia; ^5^Department of Anesthesia, Al Noor Specialist Hospital, Makkah, Saudi Arabia

**Keywords:** *Cryptococcus neoformans*, pigeon dropping, Saudi Arabia, DNA sequencing, KmerFinder

## Abstract

The prevalent variants of *Cryptococcus neoformans*, and other *Cryptococcus species* in pigeon excreta in Western Region of Saudi Arabia were studied. Ninety pigeon dropping samples were plated directly on Niger seed agar, and suspected colonies were sequenced using Illumina MiSeq. Species identification was determined using sequence read mapping to reference genomes of the two *C. neoformans* variants. In addition, sequence reads were identified using the KmerFinder tool. internal transcribed spacer 2 in the rDNA was also used for fungal barcoding of none of the *C. neoformans* species using two fungal identification databases. Phylogeny was studied using CSI Phylogeny (Center for Genomic Epidemiology, Denmark). The *C. neoformans* var. *grubii* mitochondrion and chromosome 1 reference sequences (accession numbers NC_004336.1 and CP022321.1, respectively) were used for sequence comparison and variant calling. Fifteen *Cryptococcus* isolates were isolated, 11 were identified as *C. neoformans* var. *grubii*, and 4 were found to be other opportunistic *Cryptococcus* species. Phylogeny analysis of *C. neoformans* var. *grubii* isolates showed a high degree of similarity between the *C. neoformans* isolates especially at the mitochondrial genome level. This study supports the fact that pathogenic and opportunistic *Cryptococcus* species are prevalent in domestic bird excreta which is an easy source of infection in the susceptible population.

## Introduction

*Cryptococcus neoformans* and *Cryptococcus gattii* are exogenous fungal pathogens with *C. neoformans* commonly associated with infections in immunocompromised patients, while *C. gattii* affects predominantly immunocompetent individuals (Girish Kumar et al., 2010). The sexual form of *C. neoformans*, *Filobasidiella neoformans*, is a filamentous fungus often found in pigeon excrement, while the sexual form of *C. gattii*, *Filobasidiella bacillispora*, lives mainly in certain types of trees. Most infections with *C. neoformans* consist of a lung infection. However, fungal meningitis and encephalitis, especially as a secondary infection for AIDS patients, are often caused by *C. neoformans*, making it a particularly dangerous fungus. There are several research groups focusing on the molecular determination of the number of genetically diverse subgroups within each *Cryptococcus* species. The molecular methods employed by each group to construct phylogenetic trees vary, and different methods have resulted in different numbers of subgroups. Interestingly, an association between geographic origin and certain genotypes has been observed, implying epidemiological significance of certain genotypes (Satoh et al., 2011; Chae et al., 2012a,b; Qishui et al., 2012; Wu et al., 2012).

*Cryptococcus neoformans*, the agent of cryptococcosis, had been considered a homogeneous species until 1949 when the existence of four serotypes was revealed based on the antigenic properties of its polysaccharide capsule. Such heterogeneity of the species, however, remained obscure until the two morphologically distinct teleomorphs of *C. neoformans* were discovered during the mid-1970s. The teleomorph *F. neoformans* was found to be produced by strains of serotypes A and D while *F. bacillispora* was found to be produced by strains of serotypes B and C. Latter studies revealed numerous differences between the anamorphs of the two *Filobasidiella* species with regard to their ecology, epidemiology, pathobiology, biochemistry, and genetics. Presently, the etiologic agent of cryptococcosis is classified into two species, *C. neoformans* (serotypes A and D) and *C. gattii* (serotypes B and C). Intraspecific genetic diversity has also been revealed as more genotyping methods have been applied for each serotype (Sugita et al., 2000). As a result, the number of scientifically valid species within *C. neoformans* has become a controversial issue because of the differing opinions among taxonomists as to the appropriate definition of a species.

Due to the availability and affordability of better-resolution molecular biology techniques, such as DNA sequencing, more reports of infections with previously less reported *Cryptococcus* species become more evident (Yong et al., 2016; Pakshir et al., 2019; Mirpourian et al., 2021). Other less common opportunistic *Cryptococcus* species are becoming an important cause of serious infections such as peritonitis and fungaemia in immunocompromised patients (Ragupathi and Reyna, 2015; Aghaei Gharehbolagh et al., 2017; Choe et al., 2020; Rodriguez-Leguizamon et al., 2020). These infections might not be an emerging infection; however, the availability of whole-genome sequence data and the availability of easy-to-use bioinformatics tools are helping medical microbiology laboratories to better detect and identify these opportunistic *Cryptococcus* species (Vajpeyi and Chandran, 2016).

The aim of this study is to identity the prevalent cryptococcus variants of *C. neoformans* in pigeon dropping in Makkah Region in Saudi Arabia using DNA sequencing. No whole-genome sequence data are currently available from isolates from this region, and therefore, nothing is known about circulating species and genotypes. Knowing the prevalent genotypes will help in the understanding of *Cryptococcus* virulence, pathogenesis, and susceptible population. In this study, the natural ecological niches of *C. neoformans* were explored and isolated species was studied by DNA sequencing. Molecular data of *C. neoformans* from our region will help in defining the molecular epidemiology and taxonomy of this important opportunistic fungal species.

## Materials and Methods

### Specimen Collection and Yeast Isolation

Specimens were collected from pigeon nets and pigeons’ feeding areas, where fresh and dry pigeons’ droppings are available in big quantities. Ninety pigeon dropping specimens were collected from the Makkah region including specimens from Makkah Holy City, Taif, and Jeddah. Specimens were collected in 30-ml sterile plastic containers and stored at room temperature. One gram of each specimen was suspended in sterile physiological saline supplemented with chloramphenicol at a final concentration of 0.1% (w/v). Serial 10-fold dilutions, in the same physiological solution, were prepared and used for direct isolation of *Cryptococcus* species. Culture was done on selective Sabouraud Dextrose Agar and Niger Seed agar and incubated for 3–5 days at room temperature. Capsulated pigmented (melanin producing) yeast colonies on Niger seed agar were presumptively identified as *Cryptococcus*. These types of yeast colonies were purified by subculture in Sabouraud Dextrose Agar, and India Ink staining was used to screen for capsule production. Yeasts colonies were purified and stored at 20% glycerol medium at −20°C.

### DNA Extraction and Genome Sequencing

Yeast cells from fresh cultures were used for DNA extraction. Cells were harvested from 2- to 3-day-old cultures and washed using sterile Tris-EDTA buffer (TE) pH 8.0 in 2-ml screw cap tubes and then resuspended in 500 μl TE buffer. The cell wall was disrupted using 0.5-mm glass beads in BioSpec Mini-Beadbeater-16 (BioSpec Inc., Bartlesville, OK, United States) for 5 min then cooled in ice for an additional 5 min. The aqueous layer containing DNA was separated from proteins and cell debris using two phenol/chloroform (1:24 pH 8.0) extractions. DNA was then precipitated by isopropanol, washed with 70% ethanol, dried at room temperature, and resuspended into 35 μl TE buffer pH 8.0. The quantity and quality of the isolated DNA were determined using Qubit^®^ (Invitrogen, Applied Biosystems, Carlsbad, CA, United States) and Agilent 2100 Bioanalyzer using DNA 1000 Chip (Agilent Inc., Santa Clara, CA, United States).

### Library Preparation for DNA Sequencing

Genomic DNA libraries were prepared using Illumina Nextera XT Library Preparation Kit, and samples were barcoded using Nextera XT Index Kit (Illumina Inc., San Diego, CA, United States). DNA sequencing libraries were prepared using 1 ng input genomic DNA and validated and quantified directly without normalization using Agilent Bioanalyzer 2100 High Sensitivity DNA Chip (Agilent Inc., United States). *Cryptococcus* genomes were sequenced in Illumina MiSeq using the paired-end protocol and version-3 600-cycle kit. The quality of the paired-end sequence reads was checked by FastQC (Galaxy Version 0.72 + galaxy1) before subsequent data analysis.

### Molecular Identification

Molecular identification was done by mapping of the sequence reads to the two possible reference genomes of *C. neoformans* variants (*neoformans* and *grubii*). Mapping was done using BWA-MEM, which maps medium and long reads (>100 bp). Reference-guided mapping was performed using chromosome 1 of each *C. neoformans* variety (accession numbers NC_006670.1 and CP022321.1, respectively) as a reference genome. Mapping was performed using BWA-MEM (Galaxy Version 0.7.17.1), and BAM files were visualized by the Integrative Genomics Viewer (IGV) version 2.8.2 (Broad Institute, Cambridge, MA, United States).

Species identification was also confirmed using KmerFinder version 3.0.2 from the Center for Genomic Epidemiology. KmerFinder predicts microbial species using a fast *K*-mer algorithm (Hasman et al., 2014; Larsen et al., 2014; Clausen et al., 2018). FastQ files of all the isolated yeast were used for the *K*-mer identification.

Isolates which showed poor mapping to *C. neoformans* reference genomes or showed poor *K*-mer scores with KmerFinder were identified based on their rDNA sequences. The internal transcribed spacer (ITS) sequences were obtained by direct Illumina sequencing, and species were identified using the UNITE ITS Database^1^ and the ISHAM ITS Database^2^. To get the most reliable identification, only the ITS 2 sequences were used for species identification.

### Phylogenetic Analysis

Phylogeny was studied using CSI Phylogeny (Center for Genomic Epidemiology, Denmark). CSI Phylogeny calls SNPs, filters the SNPs, does site validation, and infers a phylogeny based on the concatenated alignment of the high-quality SNPs (Delcher et al., 2002; Li and Durbin, 2009; Li et al., 2009; Price et al., 2010; Quinlan and Hall, 2010; Kaas et al., 2014). The *C. neoformans* var. *grubii* mitochondrion and chromosome 1 reference sequences (accession number NC_004336.1 and CP022321.1, respectively) were used for sequence comparison and variant calling.

## Results

**FIGURE 1:**
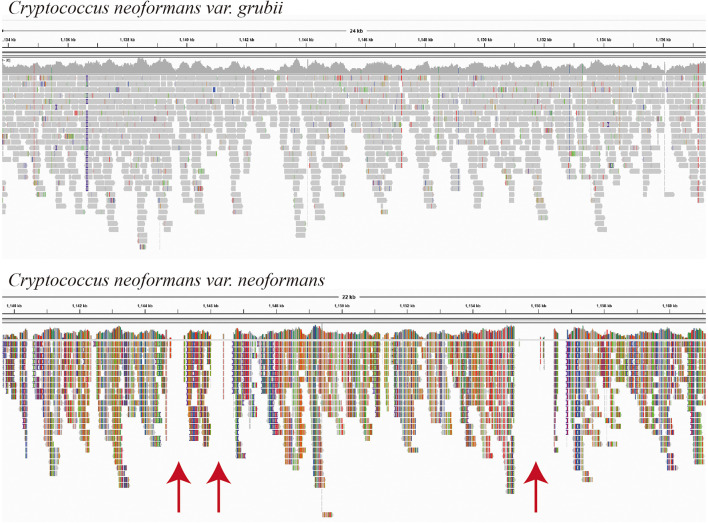
Mapping of isolate C2 sequence reads as visualized by the Integrative Genomics Viewer (IGV) version 2.8.2 (Broad Institute, Cambridge, MA, United States). Sequence reads of isolate C2 were mapped using BWA-MEM, which maps medium and long reads (>100 bp). Reference-guided mapping was performed using chromosome 1 of each *Cryptococcus neoformans* variety (accession numbers NC_006670.1 and CP022321.1, respectively) as a reference genome. Mapping was performed using BWA-MEM (Galaxy Version 0.7.17.1), and BAM files were visualized by the IGV, which show even template coverage in the case of *Cryptococcus neoformans* var. *grubii*, while in the case of *Cryptococcus neoformans* var. *neoformans* you see clear gabs in the template coverage indicated by red arrows.

Fifteen *Cryptococcus* isolates were isolated, and the yeast species identity was confirmed using DNA whole-genome sequencing with low sequence coverage (×20 to ×30) enough to map the sequence reads to the possible reference genomes. The sequence mapping showed that the closer genome for the majority of the isolates (C1–C11, 73%) was *C. neoformans* var. *grubii* (Figure 1). Mapping showed even template coverage in case of *C. neoformans* var. *grubii*, while in case of *C. neoformans* var. *neoformans* the reference genome coverage was not complete with many gabs, as shown in Figure 1.

The species identification of *C. neoformans* var. *grubii* was also confirmed by the KmerFinder tool. High *K*-mer matching scores were found with all *C. neoformans* var. *grubii* chromosomes. The chromosome coverage ranged from 86 to 93%, and the sequencing depths ranged from ×15 to ×21 (Table 1). The *K*-mer matching score showed 100% template coverage and very high sequencing depth in case of the mitochondrial genome of *C. neoformans* var. *grubii* (Table 1).

**TABLE 1:** Results of identification of *Cryptococcus* isolate number C2 using KmerFinder bioinformatic tool.

# Assembly	*K*-mer matching score	Template coverage %	Depth	Reference sequences
GCF_000149245.1	1,402,196	94	17	*Cryptococcus neoformans* var. *grubii* H99 chromosome 1
GCF_000149245.1	1,249,263	93	21	*Cryptococcus neoformans* var. *grubii* H99 chromosome 2
GCF_000149245.1	922,974	93	16	*Cryptococcus neoformans* var. *grubii* H99 chromosome 3
GCF_000149245.1	650,005	90	16	*Cryptococcus neoformans* var. *grubii* H99 chromosome 4
GCF_000149245.1	1,033,422	92	15	*Cryptococcus neoformans* var. *grubii* H99 chromosome 5
GCF_000149245.1	854,952	93	16	*Cryptococcus neoformans* var. *grubii* H99 chromosome 6
GCF_000149245.1	820,242	91	16	*Cryptococcus neoformans* var. *grubii* H99 chromosome 7
GCF_000149245.1	798,403	91	15	*Cryptococcus neoformans* var. *grubii* H99 chromosome 8
GCF_000149245.1	707,589	91	16	*Cryptococcus neoformans* var. *grubii* H99 chromosome 9
GCF_000149245.1	637,173	90	16	*Cryptococcus neoformans* var. *grubii* H99 chromosome 10
GCF_000149245.1	903,131	91	16	*Cryptococcus neoformans* var. *grubii* H99 chromosome 11
GCF_000149245.1	458,861	88	16	*Cryptococcus neoformans* var. *grubii* H99 chromosome 12
GCF_000149245.1	464,331	86	16	*Cryptococcus neoformans* var. *grubii* H99 chromosome 13
GCF_000149245.1	581,666	89	17	*Cryptococcus neoformans* var. *grubii* H99 chromosome 14
GCF_000149245.1	1,707,609	100	1809	*Cryptococcus neoformans* var. *grubii* H99 mitochondrion

*The “*K*-mers Matching Score” is the total number of matching *K*-mers between the query and the template. The “Template Coverage” is the percentage of the coverages of the reference sequences, while the “Depth” is the number of matched *K*-mers in the query sequence divided by the total number of *K*-mers in the template. Like in this case, when using read files “Depth,” this estimates the sequencing depth.*

**FIGURE 2:**
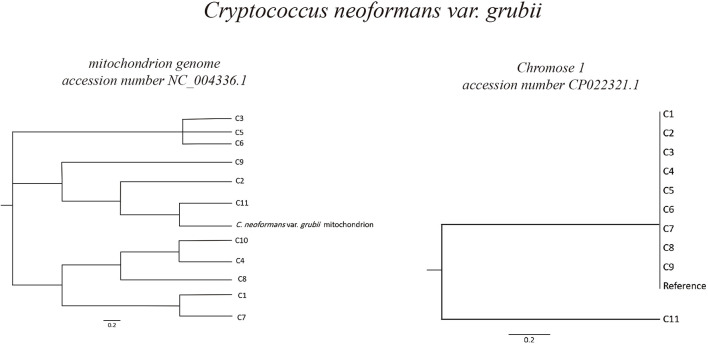
Phylogeny of *Cryptococcus neoformans* var. *grubii* isolates. Sequence read analysis was done using CSI Phylogeny (Center for Genomic Epidemiology, Denmark). CSI Phylogeny calls SNPs, filters the SNPs, does site validation, and infers a phylogeny based on the concatenated alignment of the high-quality SNPs. The *Cryptococcus neoformans* var. *grubii* mitochondrion and chromosome 1 reference sequences (accession number NC_004336.1 and CP022321.1, respectively) were used for sequence comparison and variant calling. Phylogeny analysis showed high degree of similarity of most of the isolates (10 out of 11) to *Cryptococcus neoformans* var. *grubii* chromosome 1, while analysis based on sequences comparison to the mitochondrion genome showed varied degrees of similarities.

Based on *C. neoformans* var. *grubii* chromosome 1 (accession number CP022321.1), phylogeny analysis showed high degree of similarity of most of the isolates, in which 10 out of 11 were clustered in the same branch together with the reference chromosome sequence. However, in case of phylogeny based on the *C. neoformans* var. *grubii* mitochondrion genome (accession number NC_004336.1), different isolates were found to be clustering in different branches in the phylogenetic tree (Figure 2).

Four out of the 15 isolates were not identified as *C. neoformans*. Sequencing of the ITS 2 identified these isolates as *Naganishia albidus* (formerly, *Cryptococcus albidus*) *Naganishia diffluens*, *Naganishia liquefaciens*, *Naganishia albidosimilis*, and *Naganishia globose*, which are known opportunistic *Cryptococcus* species (Table 2).

**TABLE 2:** Species identification using the UNITE ITS database and ISHAM ITS database.

Isolate #	Unite database	ID %	ISHAM database	ID%
C12	*Naganishia albida*	96.8	*Cryptococcus albidus*	93.6
	*Naganishia diffluens*	96.8		
	*Naganishia liquefaciens*	96.8		
	*Naganishia adeliensis*	96.8		
C13	*Naganishia albida*	95.8	*Cryptococcus albidus*	90.4
	*Naganishia diffluens*	95.8		
	*Naganishia liquefaciens*	95.8		
	*Naganishia albidosimilis*	95.8		
C14	*Naganishia albida*	98.4	*Cryptococcus albidus*	96.8
	*Naganishia diffluens*	98.4	*Naganishia diffluens*	96.8
	*Naganishia liquefaciens*	98.4		
	*Naganishia albidosimilis*	98.4		
C15	*Naganishia albida*	99.2	*Cryptococcus albidus*	96.0
	*Naganishia diffluens*	99.2	*Naganishia diffluens*	96.0
	*Naganishia liquefaciens*	99.2		
	*Naganishia albidosimilis*	99.2		
	*Naganishia globosa*	99.2		

To get the most reliable identification, only the ITS 2 sequences were used for species identification. Based on ITS 2 sequences, the closely related species were not differentiated even at the secondary structure levels. *Cryptococcus albidus is a synonym of Naganishia albida.

## Discussion

Many studies have recently used the whole-genome sequence data of *Cryptococcus* species to better understand the evaluation, genotype relations, diversity, pathogenicity, and antifungal susceptibility of this important genus and to explore other none *Cryptococcus* yeasts associated with pigeon dropping (Heitman et al., 1999; Florio et al., 2011; Gillece et al., 2011; Engelthaler et al., 2014; Meyer, 2015; Movahed et al., 2015; Lockhart et al., 2016). Some of these studies were carried out on *C. neoformans* and its sister species *Cryptococcus gattii*, which is an emerging pathogen connected with the ongoing cryptococcosis outbreak on Vancouver Island (Meyer, 2015; Lockhart et al., 2016).

In this study, we screened 80 environmental samples representing different areas in the Western Region in Saudi Arabia. We managed to sequence the whole genome of all positive cultures (number 15 isolates) as the only data of its type from this region. The sequence data allowed the identification of the less common opportunistic *Cryptococcus* species, confirming the variety of the pathogenic *C. neoformans* as *C. neoformans* vari. *grubii*.

As expected, *C. neoformans* was easily isolated by direct culture of pigeon dropping (Chae et al., 2012a,b; Soltani et al., 2013; Ellabib et al., 2016; Ghaderi et al., 2019; Pakshir et al., 2019; Mirpourian et al., 2021) and identification was confirmed by DNA sequencing. Sequence-based identification was confirmed by different ways. Reference genome-guided mapping of raw sequences is one way of the well-known approaches for species identifications of unknown sequence reads. Most of the isolates showed good even mapping without gabs when using the *C. neoformans* var. *grubii* H99 chromosome 1 reference sequence, while less template coverage was observed when using *C. neoformans* var. *neoformans* H99 chromosome 1 reference sequence (Figure 1). In addition, species was also confirmed using the KmerFinder which gave high scores of *K*-mer matching with chromosomal and mitochondrial genomes of *C. neoformans* var. *grubii*.

The analysis of the whole-genome sequence data of none of the *C. neoformans* isolates (n = 4) showed low scores of matching *K*-mers. These four isolates that showed no matching of *K*-mers with the fungal database were identified based on ITS2 similarity as *N. albidus* (formerly, *C. albidus*) *N. diffluens*, *N. liquefaciens*, *N. albidosimilis*, and *N. globose*. The identification of these less common opportunistic *Cryptococcus* species is becoming much easier with the availability and affordability of PCR and/or ITS sequencing for most of microbiology laboratories (Chae et al., 2012a,b; Ellabib et al., 2016; Dou et al., 2017).

We were not able to identify the less common *Cryptococcus* species using the KmerFinder tool due to lack of genome sequence data in the KmerFinder database. *N. albidus* have a draft genome record in GenBank, which has not yet been updated in the KmerFinder database (Vajpeyi and Chandran, 2016; Yong et al., 2016). Many reports showed the clinical importance of these species as opportunistic pathogens (Ragupathi and Reyna, 2015; Aghaei Gharehbolagh et al., 2017; Choe et al., 2020; Rodriguez-Leguizamon et al., 2020); however, none of these less common *C. neoformans* has been reported from this region before.

Due to similarities of morphology and ecological niches of these environmental basidiomycetous yeasts, it is always recommended to use the DNA-based method for species identification. DNA-based identification and typing are becoming the standard method of *Cryptococcus* species identification in many laboratories (Satoh et al., 2011; Chae et al., 2012b); however, scientists should always be careful and should always use tools that allow identification of all possible pathogens.

Although DNA-based methods are becoming popular and accessible to many laboratories, less common *Cryptococcus* opportunistic species can be easily missed even if DNA sequencing is used for species identification. This is because of lack of reference genomes sequence data which is needed for identification by bioinformatics tools such as KmerFinder. The identification will be more confusing when only a few sets of markers, such as ITS region, are used.

In this study, the less common *Cryptococcus* opportunistic species were poorly identified when only ITS2 sequences were used. In four isolates, more than one species was predicted and it was not possible to differentiate between *Naganishia* species (Table 2). Identifications could be improved by adding more markers, but the best way to achieve proper identification is by updating the identification databases of tools such as KmerFinder with whole-genome sequence data of these less common opportunistic yeasts species.

We conclude that *C. neoformans* var. *grubii* is the most common variety of *C. neoformans* in pigeon drooping in Saudi Arabia. Clinical microbiology laboratories should be also prepared for other opportunistic *Cryptococcus* species, which were also isolated from the same environmental specimens. Less common opportunistic yeast species should be considered when identifying clinical isolates from susceptible patients. It is important to update the fungal identification databases with more whole-genome sequence data to improve the identification of less common opportunistic yeast species.

## Data Availability Statement

The datasets presented in this study can be found in online repositories. The names of the repository/repositories and accession number(s) can be found below: https://www.ncbi.nlm.nih.gov/bioproject/, PRJNA725418.

## Author Contributions

E-SK and BSir performed all of the laboratory work. AA, HF, E-SK, and BSir contributed to conception and design of the study. MD, BSin, and MA collected all samples. All authors contributed to manuscript revision, read, and approved the submitted version.

## Conflict of Interest

The authors declare that the research was conducted in the absence of any commercial or financial relationships that could be construed as a potential conflict of interest.

## Publisher’s Note

All claims expressed in this article are solely those of the authors and do not necessarily represent those of their affiliated organizations, or those of the publisher, the editors and the reviewers. Any product that may be evaluated in this article, or claim that may be made by its manufacturer, is not guaranteed or endorsed by the publisher.

## Footnotes

1
https://unite.ut.ee/

2
https://its.mycologylab.org/
